# Efficacy and safety of Suprachoroidal Triamcinolone Acetonide in cases of resistant diabetic Macular Edema

**DOI:** 10.12669/pjms.36.2.1194

**Published:** 2020

**Authors:** Haroon Tayyab, Chaudhry Nasir Ahmed, Muhammad Ali Ayaz Sadiq

**Affiliations:** 1 Dr. Haroon Tayyab, FCPS. Al-Ehsan Welfare Eye Hospital, Lahore, Pakistan. Department of Ophthalmology, King Edward Medical University / Mayo Hospital, Lahore, Pakistan; 2 Dr. Chaudhry Nasir Ahmed, FCPS. Department of Ophthalmology, King Edward Medical University / Mayo Hospital, Lahore, Pakistan; 3 Dr. Muhammad Ali Ayaz Sadiq, FCPS. Department of Ophthalmology, King Edward Medical University / Mayo Hospital, Lahore, Pakistan

**Keywords:** Diabetic macular edema, Intravitreal, Suprachoroidal, Triamcinolone

## Abstract

**Objective:**

To evaluate the safety and efficacy of Suprachoroidal Triamcinolone Acetonide (SCTA) injection in patients with treatment resistant Diabetic Macular Edema (DME).

**Methods:**

This was a prospective non randomized interventional study conducted in the vitreoretinal department of Al Ehsan Welfare Eye Hospital, Lahore, Pakistan from March 2018 to September 2018. A total of 24 eyes of 24 patients were included. Baseline Best Corrected Visual Acuity (BCVA), Intra Ocular Pressure (IOP) and Central Subfield Thickness (CST) was recorded. After SCTA, patient was followed up at one and three months and same clinical parameters were recorded again and results analyzed.

**Results:**

Out of 24 patients, 11(45.83%) were males and 13(54.16%) were females. Mean pre injection CST was 636.5 ± 200.11 um. Mean pre injection BCVA was 0.8 ± 0.24 on ETDRS chart. Mean post injection CST at one and three months was 304.54 ± 67.43 and 302.66 ± 66.93 um. Mean post injection BCVA at one and three months was 0.47 ± 0.3 and 0.45 ± 0.27 on ETDRS chart. The results were statistically significant for pre and post injection CST at both one and three months (*p*-value < 0.00001). Pre and post injection BCVA was also statistically significant (*p*-value < 0.05).

**Conclusion:**

Preliminary evidence suggests that SCTA is well tolerated and may help in improving functional and structural outcomes of treatment resistant DME

## INTRODUCTION

Diabetic macular edema (DME) is the most common reason for loss of central vision in diabetics with diabetic retinopathy. The mainstay of DME management nowadays has changed significantly from what it was a decade ago. Lasers are no more the first line of therapy for DME.^1^ There are currently three anti VEGF agents (Eylea®, Bayer, Leverkusen, Germany - Lucentis®, Novartis, Basel, Switzerland & Avastin®, Genentech Inc., San Francisco, CA, USA) that are in use for treating DME.^2^ Despite their approval by Food and Drug Administration (FDA), not all patients are responsive to this therapy.^3^ Also, in real world situation, compliance and cost are two other factors that may lead to lesser response in DME when compared to standard controlled clinical trials.^4^ For a long time, Intra-Vitreal Triamcinolone Acetonide (IVTA) has been an alternative drug for cases not responsive to anti VEGF agents; or where compliance has been an issue. Although, IVTA has very good effect in reversing macular edema and re-establishing the compromised blood retinal barrier; its use has been plagued by certain non-desirable effects. Most notable among them is the need for repeated injections due to the waning effect of IVTA and rebound macular edema. Also, its use leads to elevated Intraocular Pressure (IOP) and cataract formation.^5^ The use of locally given steroids has proven their efficacy in certain clinical situations as first line treatment e.g., DME with psuedophakics responds equally well to steroids and ranibizumab as illustrated by Protocol I of Diabetic Retinopathy Clinical Research Network (DRCR.net).^6^ This study also reported clinically significant occurrence of elevated IOP in patients receiving IVTA.^7^ Recently, the interest in steroids has re evolved as researchers have come up with novel ways of delivering steroids in the eye. The most notable of these are Ozurdex® and Iluvein®. Ozurdex® (Allergan, Inc., Irvine, USA) is a dexamethasone implant designed to stay in vitreous cavity for six months and then biodegrade. It slowly releases the steroid in vitreous cavity. It had been approved by FDA and in most European countries for use in patients with DME. But the use of Ozurdex® is also associated with increase in IOP and it has been documented by many researchers.^8^

In pursuit of mitigating the side effects of intravitreal steroids, there has been a recent increased interest to explore suprachoroidal space for drug delivery. While choosing suprachoroidal route for steroid delivery to posterior segment, researchers have been contemplating their risk to benefit ratio accounting for the frequency of drug delivery, level of intervention and achievable posterior segment concentrations versus systemic and anterior segment concentrations.^9^ The safety and efficacy of suprachoroidal route of drug delivery has also been established in other reports as well. Getting encouraged from the recent results, we conducted a study on treatment resistant DME using Supra-Choroidal Triamcinolone Acetonide (SCTA).

## METHODS

This was a prospective non randomized interventional study conducted in the vitreoretinal department of Al Ehsan Welfare Eye Hospital from March 2018 to September 2018.(Feb. 19^th^ 2018) This study was approved by Ethical Committee of the hospital on February 19, 2018. Informed consent was taken from all the participants. A total of 24 patients were included in this study. The selection of patients was non randomized. All patients included in this study were 18 years or more of age having Type-1 or Type-2 diabetes mellitus. All patients had treatment resistant central involving DME with Best Corrected Visual Acuity (BCVA) of less than or equal to 20/40. Any patient having macular edema secondary to any other cause, IOP of more than 21 mmHg, history of previous intraocular surgery or treatment naïve patients of DME, uveitis, ocular hypertension, cataract and macular ischemia (documented on fundus fluorescein angiography) were not included in this study. Also, we excluded all those patients who had history of periocular or intravitreal triamcinolone acetonide treatment within the last 6 months.

Treatment resistance was labelled when DME failed to respond to three anti VEGF injections (any type) spaced at one month apart. Failure to respond was decided on BCVA and/or Central Subfield Thickness (CST) on Spectral Domain Optical Coherence Tomography (3D OCT-1 Maestro Spectral Domain OCT, Topcon Europe Medical BV Capelle aan den Ijssel, The Netherlands). At one month after the third anti-VEGF, if BCVA did not improve by 5 letters on Early Treatment Diabetic Retinopathy Study (ETDRS) chart or the CST did not decrease by 50um or 10% from baseline, then the case was labelled as resistant DME. These cases were then selected for SCTA injection if they met all the inclusion and exclusion criteria. At the time of initial assessment, all participants underwent complete ocular examination that included, IOP measurement (applanation method) and anterior/posterior segment examination. All patients were followed for three months after injection and their follow up visits were scheduled at one week, one month and third month after injection. At each follow-up visit, BCVA, IOP and CST was recorded for final data analysis. Main outcome measures were change in BCVA and CST from the baseline at the end of three months. All recorded data was analyzed using SPSS 20.0. We used paired t-test and Wilcoxon signed-ranked test as test of significance for normally distributed and skewed continuous data respectively. A *p*-value of 0.05 was taken as statistically significant.

### Injection technique

We used an improvised technique for SCTA injection. We used 30 gauge 1cc insulin syringe (BD Insulin Syringe with BD Ultrafine Needle; Becton, Dickinson and Company, NJ, USA). Other dispensable included 24 gauge intravenous branula and injection triamcinolone acetonide (TA) 40mg/ml (Kenakort A by GlaxoSmithKline Brentford, Middlesex, TW8 9GS, United Kingdom). All patients were dilated before SCTA and indirect ophthalmoscope was placed at hand to examine fundus after injection. Needle was withdrawn from branula and branula was cut in such a way that only 1000um of insulin syringe was exposed from the edge of branula. TA was filled in the syringe up-to the mark of 0.1 ml. The eye was painted with 10% povidone iodine solution and 5% of this solution was instilled in fornices and left there for 30 seconds. The eye was draped in a manner similar to any intraocular procedure. We marked 3.5mm from the limbus in supratemporal quadrant. After marking, 4 mg of triamcinolone acetonide (0.1ml) was injected in suprachoroidal space by inserting the needle perpendicular to sclera and bevel pointing backwards at the distance of 3.5 mm from limbus in the said quadrant. Needle was slowly withdrawn and cotton tipped applicator was applied at the site of injection to ensure minimal reflux.

Immediately after this, indirect ophthalmoscopy was performed to ensure patency of central retinal artery and to document any spillage of drug in vitreous cavity. In case, central retinal artery was found to be occluded, then anterior chamber paracentesis was performed with 15 degrees’ phacoemulsification incision knife. After the procedure, a single drop of routinely used antibiotic was instilled in the eye.

## RESULTS

**Fig.1:**
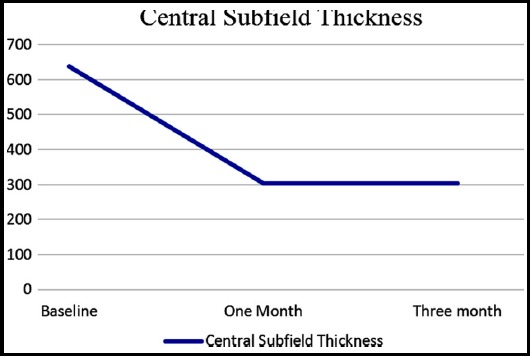
Baseline, one month and three-month post SCTA injection CST.

**Fig.2:**
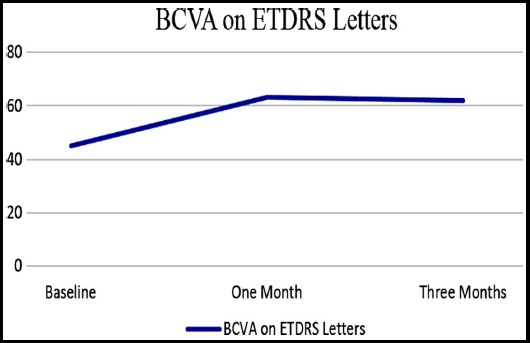
Baseline, one month and three-month post SCTA injection BCVA on ETDRS letters

A total of 24 eyes of 24 patients were enrolled in this study. Out of 24 patients, 11(45.83%) were males and 13(54.16%) were females. Mean age of the patients was 52.2917 ± 8.17 years. Mean number of previous injections received were 6.95. Maximum injections received by a patient was 11 and minimum were four. Mean pre injection and post injection (at one and 3 months) CST is shown in Fig.1. Mean pre injection IOP was 13.37 ± 2.81mmHg. Intraocular pressure at one and three months after injection was 13.95 ± 3.34 mmHg and 13.45 ± 2.32 mmHg respectively. Mean pre injection and post injection BCVA (at 1 and 3 months) is shown in Fig.2.

**Fig.3:**
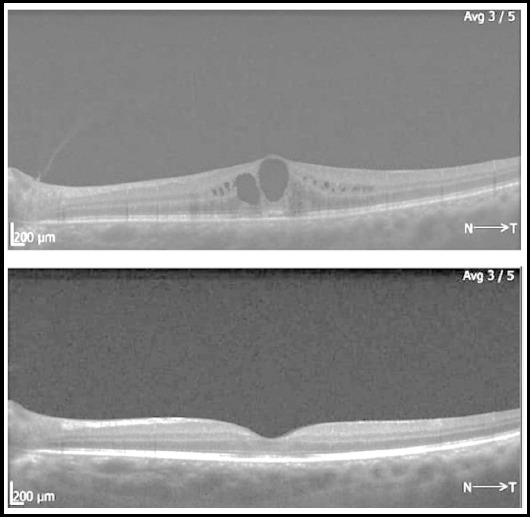
Pre and post SCTA injection SD-OCTs showing marked reduction in CST.

**Fig.4:**
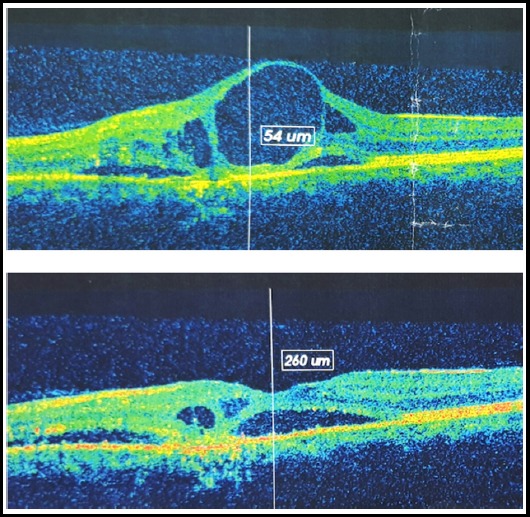
Pre and post SCTA injection SD-OCTs showing marked reduction in CST

There was statistically significant difference between pre and one-month post injection CST with p-value of < 0.00001. At three months’ post injection, the difference between pre and post injection CST was maintained with p-value of < 0.00001. The result of BCVA at three months was also statistically significant (p-value < 0.05) from the baseline. There was no difference between pre injection and post injection (one and three months) IOP when measured individually. *P*-value at one month was 0.131 and at three months was 0.711. Some of the SD-OCTs with pre injection and 3 months’ post injections results are shown in Fig.3 and 4.

There was one instance where post injection IOP rose from 19 to 24 mmHg at one month. The IOP of this patient was controlled with topical anti glaucoma therapy and returned to 16 mmHg at three months follow up. We did not encounter any other complication or unwanted sequel in our limited follow up period.

## DISCUSSION

Anti VEGF treatment for DME is a time honored strategy and standard of care nowadays. But, its use has been overshadowed by the cost involved and the number of injections required. Diabetic macular edema mandates enrollment of patients in very lengthy treatment regimens. Similarly, Ozurdex has persistently proven its efficacy in treating resistant and treatment naïve cases of DME, there has been strong evidence that it also causes elevation of IOP.^10^,^11^ According to literature, median number of injections required for DME in a single patient ranged from 9-11 in first year of treatment and mount to 17 injections over 5 years.^4^,^12^ Based on a simulated model of one year follow up of RESTORE trial, incremental cost for ranibizumab is £4,191 for 0.17 Quality Adjusted Life Year (QALY) and may reach £24,028 for estimated 15 year time.^4^ The efficacy of IVTA in treating DME is also well proven over the last many decades. But repeated studies have shown very high occurrence of cataract formation and increased IOP over time.^13^

Suprachoroidal injection of triamcinolone acetonide is a relatively newer approach for managing different retinal vascular pathologies. It has been shown in different animal models that suprachoroidal drug delivery achieves considerable posterior segment drug concentrations where as they have very low anterior chamber presence as opposed to IVTA.^14^ A recent study (HULK Trial; N = 20) found the basis of safety and efficacy of SCTA for DME in treatment naïve and previously treated groups.^15^,^16^ In case of previously treated group, mean number of injections given were 21.6 whereas in our study, mean number of previously given injections was 6.9. The difference in our study and HULK trial was that we did not include treatment naïve patients and we did not combine the first injection of SCTA with Aflibercept. The mean pre-treatment CST in previously treated arm of HULK trial was 473 um whereas in our study it was 636.5 ± 200.11 um. At six months, mean CST in HULK reduced to 369 um whereas in our study, mean CST at three month follow up was 302.66 ± 66.93 um. The difference here is the amount of follow up period that may mask any rebound of DME in our patients. Although the starting CST in HULK trial was less than in our trial, the CMT achieved at end of follow up period in our study was less than the HULK trial. In HULK study, they re-injected SCTA when needed where as we did not re-inject SCTA. In HULK study, the pre injection mean BCVA was 67.2 letters where as it was 45 letters in our study. At end of 3 months, HULK study reported a mean increase of seven letters from the baseline whereas mean increase in our study was 12 letters from the baseline. The HULK study reported continued increase in BCVA over a period of 6 months whereas we don’t have such data available in our study. The most likely reason for the apparent more letter gain in our study is that we started off with worse BCVA at base line as compared to HULK study. Also the patients enrolled in HULK study had more prolonged (21.6 vs 6.9 injections) previous treatment history which may show chronicity of disease in their patient sample. Mean IOP was 13.8 mmHg at baseline and it was 14.2 at six months of HULK trial. In our study, mean IOP at baseline was 13.37 ± 2.81mmHg and at 3 month follow up, it was 13.45 ± 2.32 mmHg. We reported a single patient where IOP rose from 19 to 24 mmHg at one month follow up that returned to 16 mmHg at three months after topical Anti Glaucoma Therapy (AGT). HULK study reported two such patients that required control of IOP with AGT. In another similar study, the authors reported no increase in IOP after SCTA. In their study, they injected nine patients with no untoward events and statistically significant reduction in macular edema.^17^

HULK report one incidence of inadvertent intravitreal spillage of triamcinolone where as we had no such incidence. Overall, the efficacy and safety of SCTA are very much comparable in both the studies despite some differences in patient selection and follow up duration.

Suprachoroidal triamcinolone injection has also been tried in Retinal Vascular Occlusion (RVO) and posterior uveitis apart from DME. In TANZANITE study, the efficacy of SCTA was compared with intra vitreal aflibercept in cases of RVO. There have been very encouraging results of TANZANITE study where combined injections have shown sustained edema resolution and improved visual outcomes with reduced number of injections.^18^ In a similar study (DOGWOOD), SCTA was used for non-infectious posterior uveitis and the authors reported satisfactory results in term of BCVA improvement and sustained reduction in CST.^19^,^20^ Similar studies (PEACHTREE Phase III trial) have been conducted to assess the safety and efficacy of SCTA.^21^

Preclinical data has shown that use of suprachoroidal space for delivery of drugs especially TA leads to their more posterior distribution with higher concentrations of drugs available for retina, choroid and retinal pigment epithelium; and lesser exposure to anterior segment. This observation potentially mitigates the known and much dreaded side effects of IVTA as shown by our and HULK study.^11^

### Limitations of the study

Shortcomings of this study include a small sample size, short duration of follow up and lack of control or comparative group. Nevertheless, suprachoroidal route of drug delivery has emerged as an effective and safe modality which may be used for other retinal pathologies apart from DME. We recommend cautious and controlled use of SCTA in selected cases and also to ensure that the surgeon first become comfortable in the technique of SCTA injection before advocating its wide spread use in multiple pathologies.
